# Smartphone‐based evaluation of awake bruxism behaviours in a sample of healthy young adults: findings from two University centres

**DOI:** 10.1111/joor.13212

**Published:** 2021-06-09

**Authors:** Alessandra Zani, Frank Lobbezoo, Alessandro Bracci, Goran Djukic, Luca Guarda‐Nardini, Riccardo Favero, Marco Ferrari, Ghizlane Aarab, Daniele Manfredini

**Affiliations:** ^1^ School of Dentistry University of Padova Padova Italy; ^2^ Department of Orofacial Pain and Dysfunction Academic Centre for Dentistry Amsterdam (ACTA University of Amsterdam and Vrije Universiteit Amsterdam Amsterdam The Netherlands; ^3^ Department of Management and Engineering University of Padova Padova Italy; ^4^ Department of Dentistry and Maxillo‐facial surgery Hospital of Treviso Treviso Italy; ^5^ School of Dentistry University of Siena Siena Italy

**Keywords:** awake bruxism, bruxism, ecological momentary assessment, prevalence, smartphone

## Abstract

A smartphone‐based ecological momentary assessment (EMA) strategy was used to assess the frequency of awake bruxism behaviours, based on the report of five oral conditions (ie relaxed jaw muscles, teeth contact, mandible bracing, teeth clenching and teeth grinding). One hundred and fifty‐three (*N* = 153) healthy young adults (mean ± SD age = 22.9 ± 3.2 years), recruited in two different Italian Universities, used a dedicated smartphone application that sent 20 alerts/day at random times for seven days. Upon alert receipt, the subjects had to report in real‐time one of the above five possible oral conditions. Individual data were used to calculate an average frequency of the study population for each day. For each condition, a coefficient of variation (CV) of frequency data was calculated as the ratio between SD and mean values over the seven recording days. Average frequency of the different behaviours over the seven days was as follows: relaxed jaw muscle, 76.4%; teeth contact, 13.6%; mandible bracing, 7.0%; teeth clenching, 2.5%; and teeth grinding, 0.5%. No significant differences were found in frequency data between the two University samples. The relaxed jaw muscles condition was more frequent in males (80.7 ± 17.7) than in females (73.4 ± 22.2). The frequency of relaxed jaw muscles condition over the period of observation had a very low coefficient of variation (0.27), while for the different awake bruxism behaviours, CV was in a range between 1.5 (teeth contact) and 4.3 (teeth grinding). Teeth contact was the most prevalent behaviour (57.5–69.7). Findings from this investigation suggest that the average frequency of AB behaviours over one week, investigated using EMA‐approach, is around 23.6%.

## 1 INTRODUCTION

Bruxism is a much‐debated topic in dentistry and several other disciplines because of its multifaceted clinical relationship with many conditions and consequences.^1^ Recently, an expert panel including professionals from different specialties (ie dentists, oro‐facial pain experts, psychologists) proposed separate definitions for awake and sleep bruxism and provided suggestions on the assessment strategies.^2^ In particular, while most research so far has been focused on the approaches to evaluate sleep bruxism (SB) (ie polysomnography [PSG], electromyography [EMG])^3^, ^4^ knowledge is poor on awake bruxism (AB).

Awake bruxism is now defined as ‘*a masticatory muscle activity during wakefulness which is characterized by repetitive or sustained tooth contact and*/*or bracing or thrusting of the mandible and is not a movement disorder in otherwise healthy individuals’*.^2^ This definition embraces the concept of bruxism as a *behaviour* that is not necessarily pathological and/or has clinical consequences.^5^, ^6^ Within this premise, it is important to determine the frequency of AB in otherwise healthy individuals for comparisons with other populations, such as individuals with possible risk factors for additive bruxism (eg psychological factors, comorbid conditions) and/or with possible bruxism consequences (eg muscle fatigue and pain, tooth wear). Besides, cross‐cultural comparisons are needed to assess the influence of different social environments and living habits on bruxism behaviours. Thus, the definition of AB has implications concerning the assessment, which should possibly be more elaborated than the single‐item self‐reported strategies that were used both in adults^7^ and in children/adolescents^8^ in the past decades.

AB can be assessed with a combination of non‐instrumental (ie self‐report and clinical observation) and instrumental (ie EMG) approaches as well as with ecological momentary assessment (EMA) strategies.^2^, ^9^ EMA is an umbrella term that gathers together the possible approaches relying on the real‐time report of a behaviour or condition under study.^10^ It potentially addresses the limitations of traditional reporting methods, such as retrospective reports, single‐item diaries and questionnaires.^7^ EMA strategies allow collecting real‐time data over a certain time frame at multiple recording points during the day, close in time with the experience in the natural environment.^11^ The everyday (‘real world’) environment, in which subjects report an experience while going on with their lives, increases the representativeness and possible generalisation of these ecological findings to an individual's real life.^12^ EMA has already been proven useful in the research field to assess oral activity^13^ but EMA‐based data on AB are fragmental and limited to a few investigations on selected behaviours.^14^, ^15^, ^16^, ^17^ Also, it must be pointed out that all studies on AB are antecedent to the 2018 definition.^2^

Considering these drawbacks, a smartphone‐based strategy that was recently introduced to implement EMA in the clinical research setting was adapted to collect data on the reported frequency of all the conditions (ie teeth contacting habits, mandible bracing, teeth clenching and teeth grinding) that are potentially part of the AB spectrum.^12^, ^18^ To get deeper into this issue, an early report provided data on the frequency of the above‐described AB behaviours over one week in a sample of healthy young adults by the adoption of a dedicated smartphone application.^19^

Findings reported a 28.3% frequency of AB behaviours, with a low coefficient of variation for the report of relaxed jaw muscle condition.

Based on that, this investigation represents an extension of the first AB‐smartphone EMA paper by Bracci et al.^19^, with the aim of collecting data on a larger sample and, importantly, on study populations recruited in two different centres. To pursue this goal, this investigation was designed to assess the frequency of AB behaviours by the adoption of EMA smartphone‐based technology over one week in a sample of healthy young adults recruited in two different University Centers.

## 2 MATERIALS AND METHODS

A sample of healthy young adults underwent a one‐week recording period with a smartphone app (BruxApp, BruxApp Research®, BruxApp team Pontedera, Italy) that was specifically developed to report and monitor the frequency of awake bruxism behaviours in an individual's natural environment. Subjects were recruited among fourth, fifth and sixth year undergraduate students attending the School of Dentistry in two different Italian Universities (Padova and Siena). The main inclusion criteria were the possession of a new‐generation smartphone and a good general health, based on the absence of TMD/oro‐facial pain and/or any documented degenerative, neurological or systemic (eg rheumatological, hormonal) diseases.

All participants were asked to attend an information session with the leading investigators (A.Z., A.B. and D.M.). During that session, the aim of the study was explained and they received a pass code for a free download of the application on their smartphones. The students had already received seminars on bruxism and received an explanation on the study aims and how to use the application. The app is based on the principle of collecting self‐reported experience during everyday life (ie ‘ecological approach’) and sends sound messages at random hours during the day to alert the individual on the condition of his/her teeth and jaw muscles. People who are using the app have to answer within 5 min by touching on the smartphone display the icon that refers to the current condition of his/her jaw muscles: relaxed jaw muscles, teeth contact, mandible bracing, teeth clenching and teeth grinding. For other details on the application and the software, readers are referred to the original publication.^18^

The five conditions were explained in person to all individuals during the training sessions, also with the support of several images and videos. The conditions were defined as follows:

1. Relaxed jaw muscles: condition of perceived relaxation of jaw muscles without teeth contact;
2. Teeth contact: condition of slight teeth contact when the mouth is closed. More precisely, it was defined as the teeth contact the subject perceived when 40 µ articulating paper (Bausch Occlusion papier®; Bausch KG, Koln, Germany) is put between dental arches and the individual is asked to slightly keep the teeth in contact to retain it on site;
3. Mandible bracing: condition in which the jaw muscle stiffness or tension resembles that during teeth clenching, but without teeth contact;
4. Teeth clenching: all conditions in which teeth contacts are more marked than the above and jaw muscles are kept tense;
5. Teeth grinding: condition in which the opposite teeth are gnashed or ground, regardless of the intensity and direction of antagonist teeth contacts;

After the explanations, the students downloaded the app and were instructed to start the first session of data collection the morning after. The software is set to send 20 alerts per day at random intervals and, based on a previous publication on the expected compliance,^20^ the participants were asked to give at least 60% of valid answers/day (ie the answer must be given within five minutes, otherwise an error message appears on the display). Days with a compliance <60% were automatically discarded. The participants were taught to answer the alert by tapping the display within this time window from the alert sound. Data were recorded over a 7‐day period, and the recording time was set from 8.00 to 12.30 and from 14.30 to 22.00. In order to have an as long as possible window‐time recording, only lunch time was excluded and the subjects were instructed to ignore alerts during meals and particular activities (ie singing). In case of failure to reach of the minimum of valid answers per day, the software automatically sets another day of recording to complete the 7‐day protocol. After seven valid days, the software generated an anonymous.csv file that the participants sent to the researchers via a dedicated email.

A descriptive evaluation of the frequency of each condition, calculated as a percentage with respect to the answered alerts, was performed in all individuals. The frequency was calculated on individual basis, and individual frequencies were used to calculate an average of the study population on a daily basis. At the end of the 7‐day collecting period, the mean frequency of each condition was assessed both for each subject and for the entire study population. Data were reported as mean values of the 7‐day span per condition according to the strategy of reporting EMA data described by Kaplan and Ohrbach^13^ and Bracci and colleagues.^19^ For each condition, a coefficient of variation (CV) of frequency data was assessed as the ratio between SD and mean values over the seven recording days.

Between‐gender comparison was performed by using t test for unpaired data, with significance level set at *p* < .05. The same significance level was used for between‐universities population comparison. In addition, as a second analysis, the prevalence values of each behaviour on a subject level, viz., the proportion of subjects indicating the behaviour at least one time, were determined for each day.

The data were collected by the leading author of this manuscript (A. Z.) in collaboration with two undergraduate dental students (one for each University) who are following the smartphone‐EMA bruxism project as part of their dental degree thesis. All statistical procedures were performed with the software SPSS 25.0 (IBM, Milan, Italy).

The study protocol was approved by the Treviso Hospital's IRB (code #344‐CES‐AULSS9) All participants signed a written consent to take part in the study.

## 3 RESULTS

All 153 students attending the final three years of the two Dental Schools were invited to take part in the study (83 from the University of Siena and 70 from the University of Padova). The sample consisted of 93 females and 60 males, with a mean age of 22.9 years (range 19–26). All students that were recruited and met the inclusion criteria completed the project. The average response rate to the alerts was 73.4 (±11.0) (Table 1), without any difference between genders. The mean compliance per day (ie percentage of alerts to which the subjects responded) was 65.8 ± 11.5% of the total alerts.

**TABLE 1:** Average response rate to the alerts (mean values and SD) over the 7‐day observation period

	D1	D2	D3	D4	D5	D6	D7	Mean of confirmed alerts
Mean	71.6	74.4	73.8	74.4	74.1	74.0	71.5	73.4
SD	15.3	15.7	16.0	14.6	13.8	14.3	16.0	11.0

On average, the frequency of the different AB behaviours over the seven days was calculated as follows: relaxed jaw muscle, 76.4% (±20.8); teeth contact, 13.6% (±14.2); mandible bracing, 7.0% (±12.5); teeth clenching, 2.5% (±4.5); and teeth grinding, 0.5% (±1.7) (see Table 2). Frequency data were not different between the two University samples (Table 3). Gender‐related frequency was different for the mean of the relaxed jaw muscles condition, which was more frequent in males (80.7 ± 17.7) than in females (73.4 ± 22.2) (see Table 4).

**TABLE 2:** Frequency data expressed in percentage of positive observation (mean values, range and coefficient of variation) for the different awake bruxism (AB) behaviours over the 7‐day observation period

Activity	Mean frequency (SD)	Range	Daily mean frequency							CV
			D1	D2	D3	D4	D5	D6	D7	
Relaxed jaw muscles	76.4 (20.8)	6.5–100	72.6 (22.0)	73.4 (23.3)	75.4 (24.4)	76.7 (23.4)	78.3 (23.9)	78.2 (24.1)	79.4 (24.1)	0.27
Teeth contact	13.6 (14.2)	0–77.9	15.1 (14.8)	14.5 (15.9)	13.6 (17.7)	13.5 (16.6)	13.1 (17.9)	12.7 (17.9)	11.9 (17.6)	1.05
Mandible bracing	7 (12.5)	0–92.8	8.27 (14.1)	8.02 (13.8)	7.2 (14.1)	6.3 (13.7)	5.9 (13.1)	6.2 (14.2)	5.9 (13.6)	1.81
Teeth clenching	2.5 (4.5)	0–19.9	2.8 (9.5)	2.1 (7.7)	3.3 (8.11)	2.1 (5.0)	2.0 (6.4)	1.6 (5.8)	1.8 (5.6)	1.87
Teeth grinding	0.5 (1.7)	0–17.7	0.5 (2.2)	0.5 (2.4)	0.7 (3.0)	0.3 (2.1)	0.2 (1.9)	0.2 (2.12)	0.6 (2.9)	4.25

Abbreviations: CV, coefficient of variation; SD, standard deviation.

*Note*: Mean frequency value is the number of positive responses for each specific behaviour per reporting period. For instance, a mean of 76.4% for the condition ‘relaxed Jaw muscles’ can be interpreted as equivalent to the report of 76.4% ‘Relaxed jaw muscles’ per 100 reporting alerts, meaning that, on average, each subject answered ‘relaxed Jaw muscles’ to 76.4% of the alerts, generalising from the random EMA sampling, with a minimum of 6.5% by at least 1 subject and a maximum of 100% by at least one subject.

Coefficient of variation (CV) is expressed as the ratio between SD and mean values of frequency data over the 7 recordings day for each condition.

**TABLE 3:** Frequency data expressed in percentage over one week. Comparison between the two University population was performed with t test for unpaired data (*p* < .05)

Activity	Siena (*n* = 83)	Padova (*n* = 70)
	Mean frequency (SD)	Mean frequency (SD)
Relaxed jaw muscles	78.8 (19.8)	73.3 (21.8)
Teeth contact	12.0 (12.7)	15.3 (15.8)
Mandible bracing	5.3 (9.2)	8.7 (15.4)
Teeth clenching	2.6 (4.7)	2.0 (4.2)
Teeth grinding	0.4 (1.2)	0.5 (2.5)

**TABLE 4:** Frequency data expressed in percentage over one week. Gender comparison performed with a t test for unpaired data (*p *< .05)

Activity	Mean Frequency (SD)	
	M (60)	F (93)
Relaxed Jaw Muscles	80.7 (17.7)	73.4 (22.2)
Teeth contact	13.2 (14.6)	13.7 (14.1)
Mandible bracing	4.0 (6.1)	8.7 (15.0)
Teeth clenching	1.8 (3.7)	2.7 (4.9)
Teeth grinding	0.25 (0.84)	0.6 (2.1)

The frequency of the relaxed jaw muscles condition was stable over the one‐week span, with a very low coefficient of variation (CV, 0.27), while CV was higher for the different AB behaviours: ‘Teeth contact’ (1.05), ‘Mandible bracing’ (1.81), ‘Teeth clenching’ (1.87) and ‘Teeth grinding’ (4.25) (Table 2).

As for the percentage of subjects reporting the different AB behaviours at least one time during the observation period, data showed that ‘teeth grinding’ was the least prevalent condition (range over the 7 days: 1.3%–6.6%). Teeth contact was the most prevalent behaviour, with a 69.7% prevalence of individuals reporting it on day 1 (range over the 7 days: 57.5%–69.7%) (Table 5).

**TABLE 5:** Prevalence data expressed as the proportion of individuals that reported the behaviour at least once a day (%)

Activity	Daily prevalence over 1 week (95% CI)						
	D1	D2	D3	D4	D5	D6	D7
Teeth Contact	69.7	65.4	59.5	63.4	62.1	57.5	59.2
Mandible bracing	46.1	48.4	39.2	32	32.7	32	31.6
Teeth clenching	20.4	18.3	22.2	20.3	17	14.4	12.5
Teeth grinding	6.6	6.5	6.5	3.9	1.3	2.6	5.3

## 4 DISCUSSION

This study provides information on the frequency of different AB behaviours by the adoption of the EMA approach. Thanks to the use of smartphone technology, which takes advantage of a tool that is already part of the daily life for a large percentage of the population,^11^, ^21^ the ecological evaluation was well accepted by all individuals, with a mean compliance of over 70% of answered alerts.

The frequency of five specific conditions (ie relaxed jaw muscles, teeth contact, mandible bracing, teeth clenching and teeth grinding) was reported over a 7‐day observation period. Such an approach allowed collecting a huge amount of data, with a total of more than 21 thousands alerts that should be answered with self‐report of the condition in real time (up to 20 alerts × 153 participants x 7 days). Findings of this study are hard to compare with other studies due to the different study designs (ie most studies are retrospective) and the commonly used strategy to collect self‐reported data at single time points.^7^ The massive data collection is a feature of all EMA observational studies and may help setting a reference value for EMA‐based frequency of AB behaviour as well as comparing findings with single‐item reports related to dietary or smoking habits, medication usage, psychological issues and comorbid conditions.

Results show that in a population of healthy young students, the most frequent AB behaviour over the 7‐day observation was teeth contact (13.6%), while the least frequent was teeth grinding (0.5%). The frequency of the reported condition of relaxed jaw muscles was 76.4%, thus indicating that the combined frequency of the different AB behaviours was 23.6%. Due to the absence of reference values for the EMA‐based frequency of AB behaviours in the literature, these findings could be seen as a reference point for future investigations on the epidemiological features of AB in healthy young adults.

As far as gender differences are concerned, the average frequency of the relaxed jaw muscles answer was more frequent in males than in females. This finding might be explained by the potential importance of psychological factors in the aetiology of AB and the assumption that females are more prone to suffer from stress and have a more emotion‐focused coping style than males.^22^ Nonetheless, it must be remarked that a previous systematic review did not find any gender differences in the frequency of AB, but none of the included studies were based on EMA.^7^ Hence, the different methods that were used in the present study as compared to the previous investigations may explain the differences in gender‐related findings.

It is noteworthy that no significant differences were found between the two University samples (Table 3 and Table 4). This finding is of particular importance if one considers the common belief that self‐reported strategies for AB may lead to bias in the data collection due to the purported questionable validity of this approach.^23^ In the present study, an identical explanation on the way of using the application was given to both study populations, with the same supporting materials (viz., slides, images, videos) presented by the same investigators. In addition, the participants downloaded the very same version of the smartphone application, regardless of the model of smartphone they owned. This attempt to minimise dishomogeneity of information might have been instrumental to ‘calibrate’ self‐report at the individual and group level, as shown by the very similar findings on the frequency of AB behaviours in the two samples. Future studies might support the assumption that this approach, based on carefully organised training sessions, leads to a reliable EMA‐based self‐report. On the other hand, it must be underlined that the features of this study population might have positively influenced the results of all training efforts, since participants were dental students in their twenties. Factors such as age and dental training of the participants could also limit the possibility to generalise findings on the frequency data to the general population, which thus requires a further appraisal in more representative general population samples.

Interestingly, there was a very low coefficient of variation over one week for the relaxed jaw muscles condition (0.27; Table 2). This means that the frequency of reported relaxed condition in a population of healthy young adults does not change relevantly from one day to another. Indeed, the variability of report over a one‐week span mostly concerns the type of specific AB behaviour. The fact that the report of specific AB behaviours is quite variable may be explained by natural fluctuation as well as some difficulties to recognise them consistently. Such findings are nonetheless important, because they suggest that relaxed muscles can be well recognised by any individual and can be taken as the complimentary reference outcome parameter to assess the combined frequency of all the AB behaviours. Based on the low daily variability in the average frequency value of this condition, EMA strategies may be considered useful to collect reliable estimates of awake bruxism, thus reducing the influence of natural fluctuation related to the assessment of specific AB conditions. Future studies could be designed to assess the factors that are responsible for the observed variation in AB behaviours over time.

Interestingly, the use of technology and EMA has introduced a new possible way to engage subjects from a therapeutic viewpoint as well (ie ecological momentary intervention [EMI]).^9^ Data collected in the present study showed a slight decrease in the average of the AB behaviours during the one‐week assessment (Table 2). This is in agreement with the theory that being asked about a behaviour in close contextual and temporal proximity to its occurrence draws an individual's attention towards the behaviour, thereby promoting self‐awareness and potentially inducing positive changes with respect to the capability to self‐recognise and avoid the behaviour (ie EMI‐biofeedback).^24^ This hypothesis should be tested in future studies in selected populations of individuals with high frequency of AB behaviours, also as a strategy to manage possible clinical consequences such as TMJ and muscle pain. On this purpose, an interesting investigation studied the effectiveness of an email‐based recording and reminding system for limiting daytime non‐functional tooth contact in patients with TMDs. Findings suggest that such strategy may have the potential to effectively control daytime non‐functional tooth contact and could be an effective strategy for the management of TMDs.^25^

The findings of our study can be compared with the pilot study adopting a similar one‐week EMA‐report design in a smaller‐sized sample.^19^ Findings were similar, with an average frequency of relaxed jaw muscles of 71.1% and a low coefficient of variation (0.44). Thus, the hypothesis that otherwise healthy young adults with dental training have a relaxed jaw muscle condition in around seven out of 10 alerts they receive in a natural environment may be considered the starting point for any future similar investigations. Potential developments for future researches are intuitively numerous and comprise the above‐indicated evaluation of associated factors and conditions that may theoretically increase or decrease the frequency of AB behaviours (eg dietary or smoking habits, medication usage, psychological issues, comorbid conditions). In particular, these data may be added to the amount on ongoing works accounting for the 2018 bruxism definition and refining the assessment strategies.^2^, ^26^ Cross‐population comparisons are also possible, because the English version of BruxApp is adopted as a template for multi‐language translation, according to a step‐by‐step procedure led by mother‐tongue experts in the field ^27^ and used within the context of an ongoing multicentre project on bruxism epidemiology.

## 5 CONCLUSIONS

Findings from this investigation suggest that the average frequency of AB behaviours over one week investigated using EMA approach is around 23.6%, and that the most frequent condition was ‘teeth contact’, with a percentage of 13.6%. Data retrieved in the two Universities samples were similar, thus suggesting that a carefully organised training session may be instrumental to minimise reporting bias. Similarly, the very low coefficient of variation over one week for the relaxed jaw muscles condition of (0.27) supports the use of smartphone‐based EMA strategies as a promising tool to collect self‐reported data for cross‐cultural and cross‐population comparisons.

## CONFLICT OF INTEREST

Authors D.M. and A.B. took part as non‐paid advisors to the development of the BruxApp software. Other Authors do not have conflict of interests concerning this investigation.

Data subject to third party restrictions.

## AUTHOR CONTRIBUTIONS

A.Z.: conceptualised the study, collected data and drafted the manuscript, primary author and principal investigator; F.L.: co‐supervised all phases of the study and revision of the manuscript; A.B.: supervised data collection and data analysis and interpretation; G.D.: co‐supervised data collection and data analysis; L.GN.: co‐supervised the study; R.F.: co‐supervised the study; M.F.: co‐supervised the study and data analysis; G.A.: co‐supervised the study and revision of the manuscript; D.M.: conceptualised the study and supervised all phases of the study and revision of the manuscript.
